# Assessment of Congenital Cytomegalovirus Prevalence Among Newborns in Minnesota During the COVID-19 Pandemic

**DOI:** 10.1001/jamanetworkopen.2022.30020

**Published:** 2022-09-02

**Authors:** Mark R. Schleiss, Sondra Rosendahl, Mark McCann, Sheila C. Dollard, Tatiana M. Lanzieri

**Affiliations:** 1 Division of Pediatric Infectious Diseases, University of Minnesota Medical School, Minneapolis; 2 Newborn Screening Program, Public Health Laboratory, Minnesota Department of Health, St Paul; 3 Division of Viral Diseases, National Center for Immunization and Respiratory Diseases, US Centers for Disease Control and Prevention, Atlanta, Georgia

## Abstract

This cross-sectional study assesses the prevalence of congenital cytomegalovirus infection among newborns screened in Minnesota before and during the COVID-19 pandemic.

## Introduction

Congenital cytomegalovirus (cCMV) infection occurs in an estimated 4.5 per 1000 live births in the US, and substantial racial and ethnic differences in prevalence have been observed.^1,2^ In February 2016, we initiated a newborn CMV screening study at 6 well-baby newborn nurseries and 3 neonatal intensive care units in Minnesota.^1^ This study was halted in April 2020 because of the COVID-19 pandemic. Here we report on cCMV prevalence in the months after study enrollment resumed.

## Methods

This cross-sectional study was approved by the institutional review boards of participating institutions (eFigure and eMethods in the Supplement).^1^ Written informed consent was obtained from the parents of all participants. The study followed the STROBE reporting guideline.

Between February 2016 and December 2021, newborns were screened for cCMV using a saliva specimen collected within 2 weeks of birth and a dried blood spot (DBS) collected for routine newborn screening. Urine was collected within 3 weeks of birth to confirm saliva or DBS positive results.

We calculated cCMV prevalence as the number of newborns with confirmed infection (by urine testing) divided by the number of newborns screened, with 95% CIs using log-binomial regression models. We compared cCMV prevalence before (April 2016 to March 2020) and during (August 2020 to December 2021) the pandemic and by data on birth year, hospital, maternal age, self-reported maternal race or ethnicity (to assess racial and ethnic differences in prevalence), and birth order collected from electronic medical records. The Wald χ^2^ test with 2-sided significance (*P* < .05) was used for all analyses, without adjustment for multiplicity. We present prevalence ratios with 95% CIs. All analyses were performed with SAS, version 9.4 (SAS Institute Inc).

## Results

This study included 19 919 newborns screened for cCMV infection before (15 697 [79%]) and during (4222 [21%]) the COVID-19 pandemic (Table 1). We confirmed cCMV infection in 70 (4.5 [95% CI, 3.5-5.6] per 1000) and 6 newborns (1.4 [95% CI, 0.6-3.2] per 1000), respectively (Table 2). Of 76 newborns with cCMV, 65 (86%) were born to mothers aged 25 years or older; however, prevalence was highest among newborns of mothers aged 24 years or younger (6.0 [95% CI, 3.3-10.7] per 1000). Sixty-two newborns with cCMV (82%) had non-Hispanic White mothers, with a prevalence (4.6 [95% CI, 3.6-5.8] per 1000) comparable with that of non-Hispanic Black mothers (5.1 [95% CI, 2.7-9.8] per 1000). Prevalence was higher among second (6.0 [95% CI, 4.4-8.3] per 1000) compared with first newborns (3.2 [95% CI, 2.2-4.7] per 1000).

**Table 1.** Characteristics of Newborns Screened for Congenital Cytomegalovirus Infection Before and During the COVID-19 Pandemic in Minnesota, 2016 to 2021

Characteristic	No. of newborns screened (%)	
	Prepandemic (n = 15 697)^a^	During pandemic (n = 4222)
Hospital		
A	3160 (20)	814 (19)
B	2527 (16)	756 (18)
C	4198 (27)	1031 (24)
D	4216 (27)	874 (21)
E	1596 (10)	209 (5)
F	0	538 (13)
Mother’s age group, y		
≤24	1435 (9)	411 (10)
25-29	3480 (22)	952 (23)
30-34	6667 (42)	1716 (41)
≥35	4115 (26)	1140 (27)
Unknown	0	3 (0.1)
Mother’s race or Hispanic ethnicity		
Hispanic^b^	1408 (9)	271 (6)
Non-Hispanic		
Black	1390 (9)	374 (9)
White	11 069 (71)	2531 (60)
Other^c^	1334 (8)	342 (8)
Unknown	496 (3)	704 (17)
Birth order		
First	6787 (43)	1660 (39)
Second	4948 (32)	1368 (32)
Third or higher	2864 (18)	891 (21)
Unknown	1098 (7)	303 (7)

^a^ The prepandemic period includes data for April 2016 to March 2020, and the pandemic period includes August 2020 to December 2021.

^b^ Includes all Hispanic individuals regardless of race or ethnicity.

^c^ Includes Alaska Native or American Indian, Asian, Native Hawaiian or other Pacific Islander, or multiple races or ethnicities.

**Table 2.** Prevalence of Congenital Cytomegalovirus Infection in Minnesota Before and During the COVID-19 Pandemic and by Selected Maternal and Newborn Characteristics, 2016 to 2021^a^

Characteristic	No. of newborns screened (%)	No. of newborns with cCMV (%)	cCMV prevalence per 1000 (95% CI)	Prevalence ratio (95% CI)
Overall	19 919 (100)	76 (100)	3.8 (3.0-4.8)	NA	
Study period^b^					
Prepandemic	15 697 (79)	70 (92)	4.5 (3.5-5.6)	1 [Reference]	
Pandemic	4222 (21)	6 (8)	1.4 (0.6-3.2)	0.3 (0.1-0.7)	
Birth year					
2016	835 (4)	1 (1)	1.2 (0.2-8.5)	0.3 (0.4-2.0)	
2017	4332 (22)	16 (21)	3.7 (2.3-6.0)	0.8 (0.4-1.5)	
2018	4838 (24)	22 (29)	4.5 (3.0-6.9)	1 [Reference]	
2019	4740 (24)	25 (33)	5.3 (3.6-7.8)	1.2 (0.7-2.1)	
2020	1509 (8)	8 (11)	5.3 (2.7-10.6)	1.2 (0.5-2.6)	
2021	3665 (18)	4 (5)	1.1 (0.4-2.9)	0.2 (0.1-0.7)	
Hospital					
A	3794 (20)	15 (20)	4.0 (2.4-6.6)	1.1 (0.5-2.3)	
B	3283 (16)	12 (16)	3.7 (2.1-6.4)	1 [Reference]	
C	5229 (26)	20 (26)	3.8 (2.5-5.9)	1.0 (0.5-2.1)	
D	5090 (26)	20 (26)	3.9 (2.5-6.1)	1.1 (0.5-2.2)	
E	1805 (9)	8 (11)	4.4 (2.2-8.8)	1.2 (0.5-3.0)	
F (only pandemic)	538 (3)	1 (1)	1.9 (0.1-13.2)	0.5 (0.1-3.9)	
Hospital nursery					
Well baby	18 669 (94)	71 (93)	3.8 (3.0-4.8)	1 [Reference]	
Neonatal intensive care	1107 (6)	5 (7)	4.5 (1.8-10.8)	1.2 (0.5-2.9)	
Mother’s age group, y					
≤24	1846 (9)	11 (14)	6.0 (3.3-10.7)	1.9 (0.9-4.2)	
25-29	4432 (22)	14 (18)	3.2 (1.9-5.3)	1 [Reference]	
30-34	8383 (42)	33 (43)	3.9 (2.8-5.5)	1.2 (0.7-2.3)	
≥35	5255 (26)	18 (24)	3.4 (2.1-5.4)	1.1 (0.5-2.2)	
Mother’s race or ethnicity					
Hispanic^c^	1678 (9)	1 (1)	0.6 (0.1-4.2)	0.1 (0.02-0.9)	
Non-Hispanic					
Black	1764 (9)	9 (12)	5.1 (2.7-9.8)	1 [Reference]	
White	13 600 (73)	62 (82)	4.6 (3.6-5.8)	0.9 (0.4-1.8)	
Other^d^	1676 (9)	4 (5)	2.4 (0.9-6.4)	0.5 (0.1-1.5)	
Birth order					
First	8447 (46)	27 (36)	3.2 (2.2-4.7)	1 [Reference]	
Second	6316 (34)	38 (50)	6.0 (4.4-8.3)	1.9 (1.2-3.1)	
Third or higher	3755 (20)	11 (14)	2.9 (1.6-5.3)	0.9 (0.5-1.8)	

Abbreviations: cCMV, congenital cytomegalovirus; NA, not applicable.

^a^ Prevalence was calculated excluding participants with missing or unknown data (143 for hospital nursery, 3 for maternal age group, 1200 for race or Hispanic ethnicity, and 1401 for birth order). Column percentages were calculated excluding individuals with missing or unknown data.

^b^ The prepandemic period includes data for April 2016 to March 2020, and the pandemic period includes August 2020 to December 2021.

^c^ Includes all Hispanic individuals regardless of race or ethnicity.

^d^ Includes Alaska Native or American Indian, Asian, Native Hawaiian or other Pacific Islander, or multiple races or ethnicities.

## Discussion

In this study, cCMV prevalence decreased substantially among newborns whose mothers were pregnant for 5 months or longer from the beginning of the COVID-19 pandemic compared with February 2016 to March 2020. Reduced daycare attendance, behavioral changes, and mitigation measures at childcare facilities (smaller class sizes, increased hand hygiene and disinfection) aimed at reducing SARS-CoV-2 transmission^3^ may have contributed to this decrease. In Minnesota, 65% of childcare facilities were closed in April 2020, and approximately one-third remained closed until the end of 2021.^4^

This study has limitations. Although there was little variation in site-specific prevalence, sites began enrollment at different time points. In addition, families in the newborn nursery with a positive SARS-CoV-2 test result during the pandemic were not approached for enrollment. However, this likely would have had little effect, because both the prevalence of cCMV among newborns as well as SARS-CoV-2 infection among persons admitted for childbirth is low.^5^

The cCMV prevalence among newborns of non-Hispanic Black and non-Hispanic White mothers (5.1 and 4.6 per 1000, respectively) was comparable in this study, in contrast with prior estimates (9.5 and 2.7 per 1000, respectively).^2^ In the US, CMV immunoglobulin G seropositivity among women 20 to 49 years is greater than 85% for non-Hispanic Black individuals and approximately 50% for non-Hispanic White individuals.^6^ Thus, with a predominately non-Hispanic White population, national seroprevalence data suggest that a substantial proportion of pregnant women in Minnesota would likely be susceptible to primary CMV infection. We also observed a higher cCMV prevalence among second-born infants. Further understanding of the potential effects of behavioral interventions to reduce CMV risk during pregnancy and of future CMV vaccination for women of childbearing age and young children is needed. Vaccination of young children may be associated with reduced CMV transmission within households and childcare centers and could potentially affect transmission rates.

## Notes

Supplement.
eMethods

eReference
**eFigure.** Monthly Enrollment in the Congenital Cytomegalovirus Newborn Screening Study in Minnesota by Site, 2016 to 2021
