# Supplementary material for: Assessment of Congenital Cytomegalovirus Prevalence Among Newborns in Minnesota During the COVID-19 Pandemic
Source: JAMA Netw Open. 2022 Sep 2;5(9):e2230020. doi: 10.1001/jamanetworkopen.2022.30020 (PMC9440402; doi:10.1001/jamanetworkopen.2022.30020)
Supplement: Supplement. — eMethods eReference eFigure. Monthly Enrollment in the Congenital Cytomegalovirus Newborn Screening Study in Minnesota by Site, 2016 to 2021 [file jamanetwopen-e2230020-s001.pdf]

Supplementary Online Content

Schleiss MR, Rosendahl S, McCann M, Dollard SC, Lanzieri TM. Assessment of congenital cytomegalovirus prevalence among newborns in Minnesota during the COVID-19 pandemic. *JAMA Netw Open*. 2022;5(9):e2230020. doi:10.1001/jamanetworkopen.2022.30020

eMethods

eReference

eFigure. Monthly Enrollment in the Congenital Cytomegalovirus Newborn Screening Study in Minnesota by Site, 2016 to 2021

This supplementary material has been provided by the authors to give readers additional information about their work.

© 2022 Schleiss MR et al. *JAMA Network Open*.

eMethods

In 2016, we established a newborn screening study to assess sensitivity of dried blood spots (DBS) for newborn screening for congenital CMV infection using saliva as the reference standard for screening and urine collected within 3 weeks of life for confirmation.[1] The study was initiated in 5 well-baby newborn nurseries and 3 neonatal intensive care units in Minneapolis/St. Paul, Minnesota, in a stepwise manner during February 2016—March 2017 (Figure 1). Study enrollment occurred on weekdays during regular business hours, and varied over time due to staffing availability. Study enrollment was halted in April 2020 due to the coronavirus disease 2019 (COVID-19) pandemic, and resumed gradually in well-baby nurseries during August 2020—May 2021, with the addition of a sixth site in January 2021, a well-baby nursery in St. Cloud, Minnesota.

Newborns whose parent(s) provided written informed consent were screened using a saliva specimen collected prior to discharge or within 2 weeks of birth, and using DBS collected between 24 and 48 hours of age as part of routine newborn screening. The study was approved by the Institutional Review Boards at the University of Minnesota (UMN), Allina Health, Minnesota Department of Health, St. Cloud Hospital, and the Centers for Disease Control and Prevention (CDC).

Our original sample size was projected at 25,000 newborns, based on the group sample sizes required to evaluate DBS clinical sensitivity, i.e., the ability of a test to identify cases of cCMV disease present at birth or that manifests by age 3 to 4 years.

Interim analysis based on 12,554 study subjects enrolled through June 2019 demonstrated a high analytical sensitivity for DBS compared to other studies that performed population-based screening. DBS PCR testing sensitivity was 73.2% (95% confidence interval [CI], 60.4-83.0%) based on results from UMN and 76.8% (95% CI, 64.2-85.9%) from CDC, whereas saliva swab testing was 93% sensitive (95% CI, 83-97%).[1]

From February 2016 to December 2021, the overall number of live births eligible for enrollment, i.e. at the hospital during recruitment hours, was 37,689, with 29,521 (78%) before the pandemic, and 8,168 (22%) during the pandemic. Among these, families were approached for enrollment of 23,373 (79%) and 6,452 (79%) newborns, respectively, and 15,697 (67%) and 4,222 (65%) newborns were screened with parental consent (eFigure 1).

Providers of screen-positive infants were notified of the result at a median of 13 days of life so that confirmatory urine CMV PCR testing could be obtained. A total of 76 (3.8 per 1,000) newborns were identified with confirmed cCMV infection; of these, 55 (72%) were DBS-positive by the UMN lab, 58 (76%) were DBS-positive based on CDC lab results, and 71 (93%) had saliva-positive results.

eReference

1. Dollard SC, Dreon M, Hernandez-Alvarado N, et al. Sensitivity of Dried Blood Spot Testing for Detection of Congenital Cytomegalovirus Infection. JAMA Pediatr 2021;175(3):e205441. DOI: 10.1001/jamapediatrics.2020.5441.

© 2022 Schleiss MR et al. *JAMA Network Open*.

eFigure. Monthly Enrollment in the Congenital Cytomegalovirus Newborn Screening Study in Minnesota by Site, 2016 to 2021

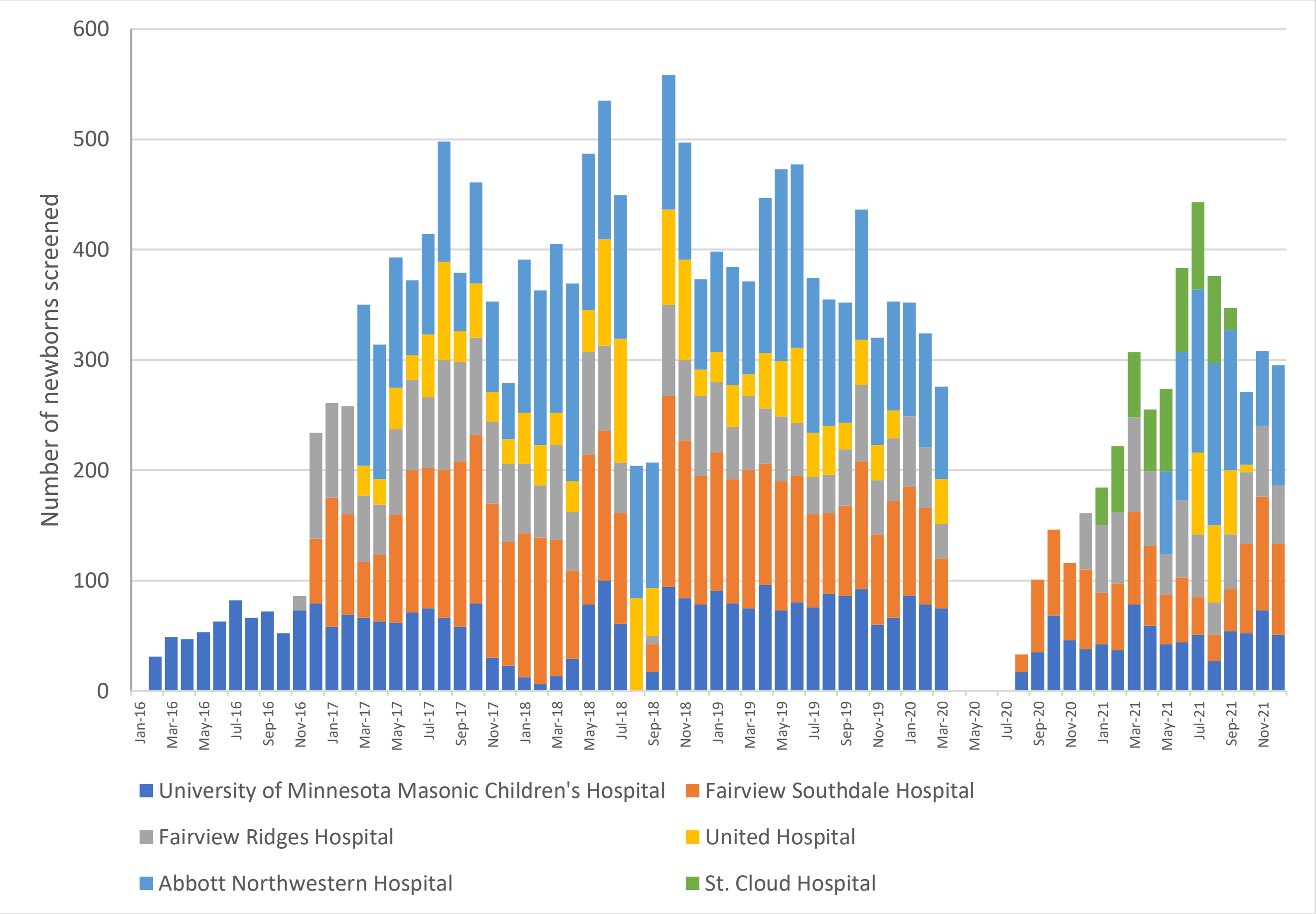

Enrollment was halted in April 2020 due to the coronavirus disease 2019 (COVID-19) pandemic and resumed gradually during August 2020—May 2021. One site was added in January 2021. The consent rate per hospital ranged from 56% to 80% before the pandemic and from 60% to 81% during the pandemic.

© 2022 Schleiss MR et al. *JAMA Network Open*.
